# c-Perpendicular Orientation of Poly(ʟ-lactide) Films

**DOI:** 10.3390/polym13101572

**Published:** 2021-05-13

**Authors:** Baku Nagendra, Paola Rizzo, Christophe Daniel, Lucia Baldino, Gaetano Guerra

**Affiliations:** 1 Department of Chemistry and Biology, INSTM Research Unit, Università di Salerno, Via Giovanni Paolo II 132, 84084 Fisciano, Italy; nbaku@unisa.it (B.N.); cdaniel@unisa.it (C.D.); gguerra@unisa.it (G.G.); 2 Department of Industrial Engineering, University of Salerno, Via Giovanni Paolo II, 132, 84084 Fisciano, Italy; lbaldino@unisa.it

**Keywords:** co-crystalline phase, α form, planar orientation, WAXD, film transparency, UV–Vis spectra

## Abstract

Poly(ʟ-lactide) (PLLA) films, even of high thickness, exhibiting co-crystalline and crystalline α phases with their chain axes preferentially perpendicular to the film plane (c_⊥_ orientation) have been obtained. This c_⊥_ orientation, unprecedented for PLLA films, can be achieved by the crystallization of amorphous films as induced by low-temperature sorption of molecules being suitable as guests of PLLA co-crystalline forms, such as *N*,*N*-dimethylformamide, cyclopentanone or 1,3-dioxolane. This kind of orientation is shown and quantified by two-dimensional wide-angle X-ray diffraction (2D-WAXD) patterns, as taken with the X-ray beam parallel to the film plane (EDGE patterns), which present all the hk0 arcs centered on the meridian. PLLA α-form films, as obtained by low-temperature guest-induced crystallization, also exhibit high transparency, being not far from those of the starting amorphous films.

## 1. Introduction

Co-crystallization of polymers with suitable guest molecules can lead, without any stretching procedure, to films (even of high thickness) with high degrees of crystal phase orientation. In fact, planar and uniplanar orientations, i.e., the preferential orientations of a crystal axis or a crystal plane with respect to the film plane, can be easily achieved [1,2,3,4,5,6,7,8,9,10,11]. Both planar and uniplanar orientations have been obtained for two commercial polymers: syndiotactic polystyrene (s-PS) [1,2,3,4,5,6,7] and poly(2,6-dimethyl-1,4-phenylene ether) (PPO) [8,9] not only for their co-crystalline forms but also for the corresponding nanoporous-crystalline phases, as produced by guest removal [10].

These planar and uniplanar orientations of co-crystalline and nanoporous-crystalline films can lead to the control of relevant properties (guest diffusivity, [6,11,12] guest orientation [13,14,15]) and can be a tool to obtain information on crystalline structures (assignment of vibrational modes of crystalline polymer chains) [16] and on crystal-to-crystal transitions [17].

Furthermore, for PLLA, a relevant commercial biodegradable polymer, crystallization as induced by sorption of several guest molecules (cyclopentanone, tetrahydrofuran, 1,3-dioxolane and *N*,*N*-dimethylformamide) in amorphous films can lead to co-crystalline phases with a high degree of orientation. In particular, a uniplanar orientation with ac-planes of crystallites being preferentially parallel to the film plane (defined as a-parallel, c-parallel, shortly a_||_c_||_) has been recently observed [18].

In the present paper, we show that suitable procedures for guest-induced crystallization on amorphous PLLA films can lead to an unprecedented co-crystalline and crystalline phase orientation with chain axes being preferentially perpendicular to the film plane (c-perpendicular orientation or more shortly c_⊥_ orientation) [2,3,4,5]. We also show that these co-crystallization procedures leading to c_⊥_ oriented films also lead to high transparency, being not far from those of starting amorphous films.

## 2. Materials and Methods

### 2.1. Materials

PLLA polymer (M_w_ ~152,000 and M_n_ ~99,000), *N*,*N*-dimethylformamide (DMF), cyclopentanone, 1,3-dioxolane, chloroform, hexane, methanol, and CaCl_2_ were supplied by Aldrich, Milan, Italy, and used as received.

### 2.2. Film Preparation

Amorphous PLLA films were obtained by melting above 180 °C, followed by rapid quenching in an ice cold water bath, films previously prepared by room temperature casting from 4 wt% chloroform solutions.

Co-crystalline films were obtained by immersion of amorphous PLLA films in various liquid solvents, which are suitable as guests of co-crystalline forms of PLLA. Most of these crystallization experiments were conducted in two different conditions: at room temperature for 30 min and at −18 °C for 72 h. α-form films were obtained from PLLA co-crystalline films by guest extraction by sorption/desorption of hexane or of methanol at room temperature.

α-form films with a lower degree of crystallinity were also obtained by cold crystallization (at ~100 °C for 30 min) of amorphous PLLA films.

### 2.3. Characterizations:

Two-dimensional wide angle X-ray diffraction (2D-WAXD) patterns were collected by a D8 QUEST Bruker diffractometer, Karlsruhe, Germany (CuKα radiation X-ray source, λ = 0.15418 nm). EDGE or THROUGH patterns were collected by sending the X-ray beam parallel or perpendicular to the film surface, respectively.

The degree of orientation of the crystalline phase, *f_c_*, was calculated by using Herman’s orientation function [9,18]:

(1) $$f_{c}=\left(\bar{3cos^{2}\gamma }-1\right)/2$$

where cos^2^ γ was calculated by the azimuthal distribution of the main reflection (110/200, at 2θ = 16.7°), for the EDGE patterns. Based on these assumptions, when *f_c_* is equal to 0, a random crystallite orientation occurs, while when *f_c_* is equal to –0.5 the c axes of all crystallites are perfectly perpendicular to the film plane.

Optical transmittance of PLLA films, with thicknesses of 25 μm and 100 μm, was measured by using a Shimadzu, Duisburg, Germany UV–Vis spectrophotometer (UV-2600). Films, after guest removal, were placed between two quartz plates, and the transmittance was measured as a function of wavelength in the 200–800 nm range.

Density of PLLA films was measured, after guest removal, by the flotation method, in aqueous solutions of CaCl_2_, at room temperature. The degree of crystallinity of the films (χ_d_) was evaluated by using the density of α crystalline phase [19,20,21]. Experimental uncertainties indicated in Table 1 were obtained by using the standard deviation method on a minimum of five measurements.

Differential scanning calorimetry (DSC) TA Q2000 equipment, New Castle, DE, USA, was used to measure the melting behavior of the PLLA films. All the measurements were made under the control heating and cooling rates (at a rate of 10 °C/min) (see Supplementary Figure S1).

Scanning electron microscopy (Carl Zeiss SMT AG, Oberkochen, Germany) was used to study the PLLA films’ surface morphology. Before imaging, all films were coated with thin gold layer (Agar Auto Sputter Coater mod. 108 A, Stansted, UK) at 30 mA for 5 min.

## 3. Results and Discussion

### 3.1. c_⊥_ Orientation of Co-Crystalline and α Phases in PLLA Films

It is well known that PLLA is able to form host–guest co-crystalline forms with suitable guest molecules like cyclopentanone, tetrahydrofuran, 1,3-dioxolane, γ-butyrolactone and *N*,*N*-dimethylformamide (DMF), [18,22,23,24,25] and some crystalline structures have also been proposed [18,22].

**Figure 1:**
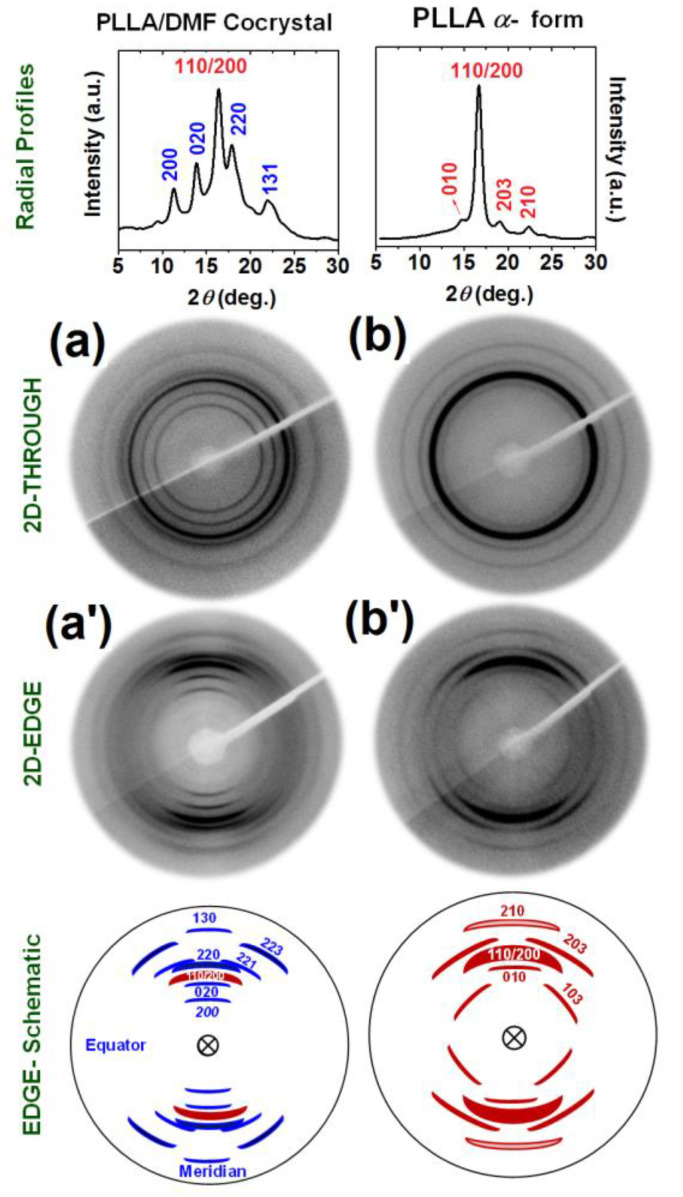
The 2D-WAXD patterns of an amorphous PLLA film, after crystallization by DMF sorption at −18 °C (**a**,**a′**) and of the same film after DMF guest removal (α form) (**b**,**b′**): (**a**,**b**) 2D-THROUGH patterns with corresponding radial profiles; (**a′**,**b′**) 2D-EDGE patterns with corresponding schematic indication of Miller’s indexes of the main reflections. In the profiles and schemes, blue and red digits indicate Miller indexes of reflections of the PLLA/DMF co-crystalline phase and of α phase, respectively.

For an amorphous PLLA film crystallized by DMF sorption at −18 °C, 2D-WAXD patterns as taken with the X-ray beam perpendicular (THROUGH pattern) and parallel (EDGE pattern) to the film plane, are shown in Figure 1a,a′, respectively. It is apparent that the THROUGH pattern (Figure 1a) exhibits only Debye’s diffraction rings, while the EDGE pattern (Figure 1a′) exhibits the diffraction arc. The rings in the THROUGH pattern indicate the absence of axial orientation, while the arcs in the EDGE pattern indicate the presence of a planar orientation.

As for the THROUGH pattern, a diffraction radial profile is shown in the upper part of Figure 1a. Close to most reflections, Miller’s indexes, based on the crystal structure of the co-crystalline PLLA/DMF form proposed in [21], are indicated by blue digits. The presence of the most intense reflection of the α form (at 2θ_CuK__α_ = 16.7°, corresponding to Miller indexes 110/200 [19,21]) indicates that the used crystallization procedure leads to the formation of the PLLA/DMF co-crystalline form as well as of a minor content of α form (≈40% of the overall crystallinity).

As for the EDGE pattern, a scheme of the diffraction arc is shown in the lower part of Figure 1a, with corresponding Miller’s indexes [22]. Centering of all the hk0 reflections along the meridian clearly indicates an orientation of the chain axes of the crystallites preferentially perpendicular to the film plane (c_⊥_ orientation). This kind of orientation has been described for co-crystalline phases of s-PS [2,3] as well as of PPO, [9] while it is unprecedented for co-crystalline phases of PLLA. The degree of c_⊥_ orientation of co-crystalline and α form phases, as evaluated from the azimuthal scans of their 020 and 110/200 reflections, is not far from *f_c_* = −0.3 for both phases.

It is worth adding that guest-induced crystallization of amorphous PLLA films, by sorption at −18 °C of cyclopentanone or 1,3-dioxolane also leads to c_⊥_ orientation of their co-crystalline phases, although with a lower degree of orientation (*f_c_* ≈ − 0.1).

The 2D-WAXD patterns of the film including a PLLA/DMF co-crystalline phase (Figure 1a,a′), after guest removal by methanol, i.e., after a treatment leading to a complete transition to the α form of PLLA, [26,27] are shown in Figure 1b,b′. As already observed for the co-crystalline film of Figure 1a,a′, the THROUGH pattern exhibits only Debye’s diffraction rings while the EDGE pattern exhibits a diffraction arc. As for the THROUGH pattern, the diffraction radial profile, with Miller’s indexes based on the crystal structure of the α form [19], is shown in the upper part of Figure 1b. As for the EDGE pattern, a scheme of the diffraction arc with corresponding Miller’s indexes is shown in the lower part of Figure 1b′. The centering of all the hk0 reflections along the meridian of the EDGE pattern again indicates the presence of a c_⊥_ orientation. The degree of the c_⊥_ orientations as evaluated by EDGE patterns of Figure 1b′ is −0.28, thus indicating that the guest removal procedure leading to α form allows nearly complete maintenance of the c_⊥_ orientation.

The degree of crystallinity of the α form PLLA film with c_⊥_ orientation of Figure 1b′, as evaluated by density measurements, is χ_d_ = 35% (2nd column of Table 1).

### 3.2. High Transparency of PLLA Films as Obtained by Low Temperature Co-Crystallization

**Figure 2:**
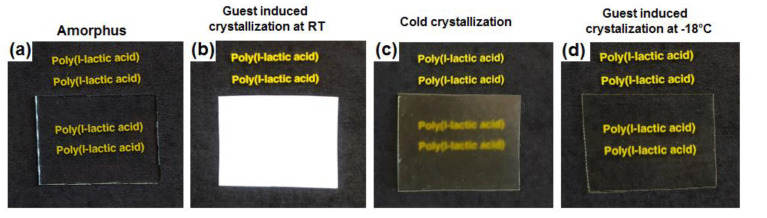
Digital photos of PLLA films with thickness of nearly 100 μm: (**a**) amorphous; (**b**) α form by DMF-induced crystallization at room temperature (unoriented with χ_d_ = 41%); (**c**) α form by cold crystallization (unoriented with χ_d_ = 28%); (**d**) α form from DMF-induced crystallization at −18 °C (c_⊥_-oriented with χ_d_ = 35%).

Photos of PLLA films with thickness of nearly 100 μm, as obtained by different procedures from amorphous films, are shown in Figure 2. As expected, the amorphous film (Figure 2a) is more transparent than all the semicrystalline α form PLLA films (Figure 2b–d).

The high transparency of fully amorphous polymer films is due to their homogeneous refractive index and is generally lost as a consequence of the formation of crystalline phases. In fact, crystalline phases typically exhibit higher density and hence a higher refractive index with respect to corresponding amorphous phases. This leads to inhomogeneous refractive indexes, which generate light scattering thus reducing the amount of light transmitted in the normal direction [28,29,30,31,32]. It is well known that suitable procedures to increase the transparency of semicrystalline polymer films generally imply the reduction of crystal size to values smaller than the visible light wavelength [28,32]. In particular for PLLA, [33,34,35,36,37,38,39] it is known that transparent semicrystalline films can be obtained by using techniques which reduce the crystallite size, e.g., CO_2_-induced crystallization in amorphous films [33,34] or addition of suitable nucleating agents [35,36]. It is worth adding that PLA film transparency has also been improved by crystallization procedures leading to the stereocomplexes between the PLLA and PDLA blends [37,38,39].

We can show that high transparency, not far from that of the starting amorphous films (Figure 2a), is obtained for α form PLLA films crystallized by DMF sorption at −18 °C, which exhibit c_⊥_ orientation (Figure 2d). These films are highly transparent, irrespective of their high degree of crystallinity (χ_d_ = 35%; Table 1).

**Figure 3:**
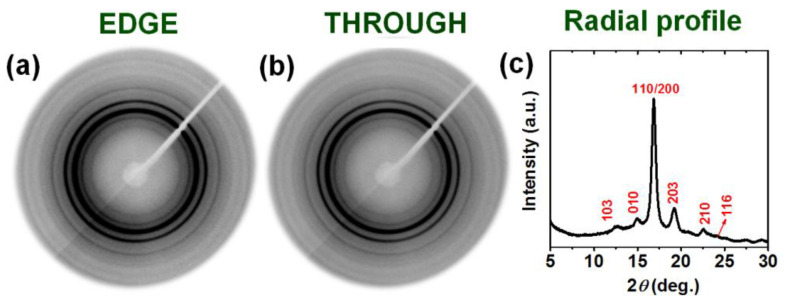
The 2D-WAXD patterns, as collected by EDGE (**a**) and THROUGH (**b**) geometries, of an amorphous PLLA film after co-crystallization by DMF sorption at room temperature followed by guest removal. Nearly indistinguishable EDGE and THROUGH patterns, only exhibiting Debye’s rings, indicate the formation of a fully unoriented α phase. (**c**) Radial profile as collected from the EDGE pattern.

The α form film, as obtained by guest-induced crystallization at room temperature, which is characterized by a high degree of crystallinity (χ_d_ = 41%) and is fully unoriented (Figure 3), is nearly completely opaque (Figure 2b).

The unoriented α form films as obtained by cold crystallization (Figure 2c) is partially opaque, although it is characterized by a low degree of crystallinity (χ_d_ = 28%).

**Figure 4:**
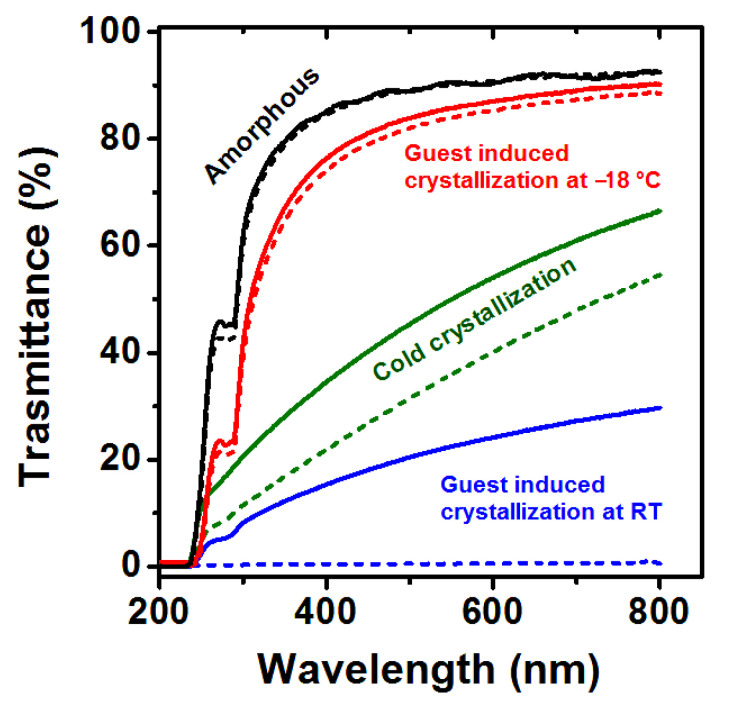
UV–Vis transmittance spectra, in the 200–800 nm range, of PLLA films: (black) amorphous; (red) α form from DMF-induced crystallization at −18 °C (c_⊥_-oriented, with χ_d_ = 35%); (green) α form from cold crystallization (unoriented, with χ_d_ = 28%); (blue) α form from DMF-induced crystallization at room temperature (unoriented, with χ_d_ = 41%). Continuous and dashed lines refer to films with thickness close to 25 µm and 100 µm, respectively.

This transparency information was quantified by UV–Visible spectra in the range 200–800 nm, for two sets of PLLA films with a thickness close to 100 µm and 25 µm, which are shown in Figure 4 as continuous and dashed lines, respectively. Transmittance values for the wavelength of 600 nm are collected in Table 1 (4th and 5th columns, for films with thickness of 100 µm and 25 µm, respectively). For instance, the transmittance of 100 µm films being amorphous, co-crystallized at low temperature, thermally crystallized, and co-crystallized at room temperature are close to 91, 86, 40, and 0%, respectively.

**Figure 5:**
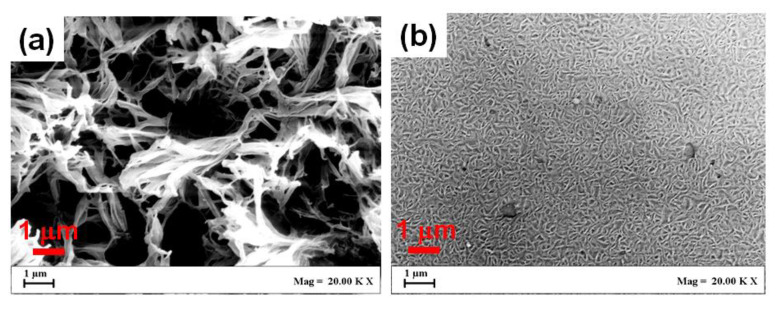
SEM images of the surface of PLLA α-form films, as obtained by DMF-guest-induced crystallization at room temperature (**a**) and at −18 °C (**b**). For both films the DMF guest was removed by sorption/desorption of methanol at room temperature.

The great differences in transparency of the PLLA α-form films as crystallized by DMF sorption at −18 °C and room temperature (followed by guest removal) can be easily rationalized by their SEM images (Figure 5a,b, respectively). In fact, the very large crystallites (interconnected lamellae, Figure 5a) obtained in the room temperature procedure were replaced in the low temperature procedure by nanometric crystallites (Figure 5b).

Film transparency, of course, is an important factor for many PLLA applications, mostly for packaging and coatings [40,41,42].

## 4. Conclusions

PLLA macroscopic films, with the orientation of the chain axes of their co-crystalline and crystalline phases being preferentially perpendicular to the film plane (c_⊥_ orientation), are reported for the first time.

These c_⊥_ oriented films are obtained by co-crystallization of amorphous PLLA films, as induced by below ambient temperature sorption of suitable guest molecules (DMF, cyclopentanone or 1,3-dioxolane).

The kind and degree of orientation was established by 2D-WAXD patterns, as taken with X-ray beam parallel to the film plane (EDGE patterns). For instance, for an amorphous PLLA film crystallized by DMF sorption at −18 °C, the degree of c_⊥_ orientation both for co-crystalline and derived α form PLLA films is not far from fc = −0.3 (*f_c_* = 0 for a random crystallite orientation, while *f_c_* = −0.5 when all crystalline chain axes are perfectly perpendicular to the film plane).

This paper also shows that α form PLLA films with c_⊥_ orientation exhibit high transparency, being not far from those of the original amorphous films. The observed behavior is rationalized by formation at low crystallization temperatures of very small polymer crystallites. High transparency of PLLA films is an important factor for packaging and coatings applications.

## Figures and Tables

**Table 1:** Density, degrees of crystallinity from density (χ_d_), and UV–Visible transmittance (at 600 nm), for PLLA films. DSC scans for the three semi-crystalline films of Table 1 are shown in Figure S1.

Films	Density (g/cm^3^) (±0.04)	χ_d_ (%) (±3%)	(%) of Transmittance (At 600 nm) for 100 μm Films	(%) of Transmittance (At 600 nm) for 25 μm Films
Amorphous	1.203	0	~91%	~91%
DMF-induced crystallization at −18 °C	1.229	35	~86%	~88%
Cold crystallization	1.225	28	~40%	~55%
DMF-induced crystallization at room temperature	1.234	41	0	~25%

## Data Availability

The data presented in this study are available on request from the corresponding author.

## Supplementary Materials

The following are available online at https://www.mdpi.com/article/10.3390/polym13101572/s1, Figure S1: DSC heating scans, at a rate of 10 °C/min, of PLLA semicrystalline films of Table 1. Amorphous PLLA films as crystallized by: (**a**) DMF sorption at −18 °C; (**b**) DMF sorption at room temperature; (**c**) cold crystallization at ~100 °C. Melting enthalpy values are shown close to the melting peaks.
