# Supplementary material for: c-Perpendicular Orientation of Poly(ʟ-lactide) Films
Source: Polymers (Basel). 2021 May 13;13(10):1572. doi: 10.3390/polym13101572 (PMC8153587; doi:10.3390/polym13101572)
Supplement: Supplementary file 1 [file polymers-13-01572-s001.zip › polymers-1202852 supplementary final.pdf]

polymers

Supplementary Material: c-Perpendicular Orientation of Poly(L-lactide) Films

Baku Nagendra, Paola Rizzo, Christophe Daniel, Lucia Baldino and Gaetano Guerra

DSC scans for the three semi-crystalline films of Table 1 are shown in Figure S1. All three films exhibit broad exothermic phenomena above Tg. Moreover, the PLLA film crystallized by DMF sorption at room temperature (Figure S1b) shows a double peak of melting. These phenomena indicate the occurrence of re-crystallization. This makes difficult evaluation and comparison of the degree of crystallinity.

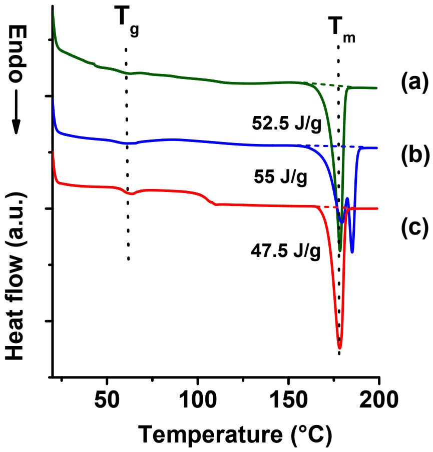

Figure S1. DSC heating scans, at a rate of 10°C/min, of PLLA semicrystalline films of Table 1. Amorphous PLLA films as crystallized by: (a) DMF sorption at -18°C; (b) DMF sorption at room temperature; (c) cold crystallization at ~100°C. Melting enthalpy values are shown close to the melting peaks.
